# Supplementary material for: Machine Learning-Based Respiration Rate and Blood Oxygen Saturation Estimation Using Photoplethysmogram Signals
Source: Bioengineering (Basel). 2023 Jan 28;10(2):167. doi: 10.3390/bioengineering10020167 (PMC9952751; doi:10.3390/bioengineering10020167)
Supplement: Supplementary file 1 [file bioengineering-10-00167-s001.zip › bioengineering-2128240-supplementary.pdf]

Supplementary Material

Table S1: Statistical Features (11 Features)

Feature Name	Description
Mean	Sum of the signal amplitude by the number of data points
Median	The value in the middle of a sorted data set
Standard deviation	Variability in the signal amplitude
Mean absolute deviation	The average distance between each data point and the mean
Signal 25th percentile feature	The value at which 25% of the signal lies below
Signal 75th percentile feature	The value at which 75% of the signal lies below
Signal's interquartile range	Difference between the 75th percentile and the 25th percentile
Skewness	It is the measure of the lack of symmetry of the signal
Kurtosis	It is the measure of the sharpness of the signal
Shannon's entropy	Entropy is a metric that evaluates the degree of randomness in the signal
Spectral entropy	The normalized Shannon's entropy is applied to the power spectrum density of the signal.

Table S2: Frequency Domain Features (27 Features)

Feature Name	Description
Maxfreq	The frequency with the highest energy
Maxval	Highest energy in the frequency domain
Maxratio	The ratio of signal energy between (f_{max} +/- delf) and the whole spectrum
24 Mel-frequency cepstral coefficients (MFCC)	Using the linear cosine transform of the log power spectrum on the nonlinear mel scale of frequency, MFCCs can be thought of as coefficients that collectively describe the short-term power spectrum of a sound.

Table S3: Time Domain Features. Each features have their mean, std and variance calculated. (45 Features)

Feature Name	Description
sys amp	Amplitude of the systolic peak of the signal
foot amp	Amplitude of the foot of the signal
x	The amplitude of systolic peak from foot (x)
t_1	Systolic peak time
t_{pi}	Time interval from one foot to other (pulse interval)
t_{pp}	Time interval from one peak to other (peak-to-peak interval)
t_1/x	The ratio of systolic peak time with systolic peak amplitude
t_1/ t_{pi}	The ratio of systolic peak time with the pulse interval
$x/(t_{pi} - t_1)$	The ratio of systolic peak amplitude with the difference between pulse interval and systolic peak time
A1	Pulse area from the beginning of beat to systolic peak
A2	Pulse area from systolic peak to the end of the beat

A1/A2	The ratio of pulse areas A1 and A2
W_25	Width of the waveform at 25% beat interval
W_50	Width of the waveform at 50% beat interval
W_75	Width of the waveform at 75% beat interval

Table S4: Derivative Features. Each features have their mean, std and variance calculated. (48 Features)

Feature Name	Description
v1	The first maximum peak amplitude from the first derivative
tv1	The first maximum peak time from the first derivative
v2	The first minimum peak amplitude from the first derivative
tv2	The first minimum peak time from the first derivative
a1	The first maximum peak amplitude from the second derivative
ta1	The first maximum peak time from the second derivative
a2	The first minimum peak amplitude from the second derivative
ta2	The first minimum peak time from the second derivative
v2/v1	The ratio of amplitudes v2 and v1
a2/a1	The ratio of amplitudes a2 and a1
tv1/tv2	The ratio of tv1 and tv2
ta1/ta2	The ratio of ta1 and ta2
tv1/ta1	The ratio of tv1 and ta1
tv1/ta2	The ratio of tv1 and ta2
tv2/ta1	The ratio of tv2 and ta1
tv2/ta2	The ratio of tv2 and ta2
